# Contrasting Photochemical Stability and Oxidative Injury Shape Drought Responses in Ferns and Mosses

**DOI:** 10.3390/plants15142143

**Published:** 2026-07-11

**Authors:** Hui Zhang, Changhui Peng, Jiahuan Guo, Qiuyu Liu, Douglass F. Jacobs, Mei Yang, Mengke Huang, Huili Feng

**Affiliations:** 1 Key Laboratory of Ministry of Education for Genetics and Germplasm Innovation of Tropical Special Trees and Ornamental Plants, School of Tropical Agriculture and Forestry (School of Agricultural and Rural Affairs, School of Rural Revitalization), Hainan University, Danzhou 571737, China; huizhang@hainanu.edu.cn (H.Z.); ym198214@hainanu.edu.cn (M.Y.); huangmengke@hainanu.edu.cn (M.H.); 2 Hainan Baoting Tropical Forest Ecosystem Observation and Research Station, School of Ecology, Hainan University, Haikou 570228, China; guojiahuan@hainanu.edu.cn; 3 Department of Biological Sciences, University of Quebec at Montreal, Montreal, QC H3C 3P8, Canada; peng.changhui@uqam.ca; 4 College of Geographic Science, Hunan Normal University, Changsha 410081, China; 5 School of Public Policy and Administration, Xi’an Jiaotong University, Xi’an 710049, China; liuqiuyu@xjtu.edu.cn; 6 Hardwood Tree Improvement and Regeneration Center, Department of Forestry and Natural Resources, Purdue University, West Lafayette, IN 47907, USA; djacobs@purdue.edu; 7 School for Forest Management, Swedish University of Agricultural Sciences, 739 21 Skinnskatteberg, Sweden

**Keywords:** drought, ferns, mosses, physiological traits, epiphytes, terrestrial ecosystems

## Abstract

Drought increasingly threatens terrestrial vegetation, whereas current syntheses remain disproportionately focused on seed plants. Ferns and mosses provide a useful contrast because they represent distinct hydration strategies among early-diverging land plants. We compiled 3272 paired observations from 46 drought experiments covering 35 fern species and 41 moss species to compare responses in water status, photosynthesis, chlorophyll fluorescence, oxidative stress, osmotic adjustment, abscisic acid, and growth. Drought reduced physiological performance in both groups, but mosses showed a greater mean decline than ferns. Ferns maintained stable maximum quantum yield and increased nonphotochemical quenching despite reduced pigment content and carbon assimilation, suggesting stronger photoprotective regulation. In contrast, mosses showed coordinated declines in maximum fluorescence, effective PSII yield, and maximum quantum yield, together with elevated minimum fluorescence, indicating direct PSII impairment. Oxidative damage, osmolyte accumulation, and growth suppression were also stronger in mosses. Within ferns, drought sensitivity was concentrated in epiphytic species, especially obligate, canopy, tank-forming, and xerophytic groups. Fern responses were partly explained by provenance climate, drought duration, and specific leaf area. Overall, within the species and experimental conditions represented in the current dataset, ferns largely maintain photochemical stability through photoregulation, whereas mosses shift more rapidly toward PSII impairment, oxidative injury, and growth suppression, highlighting vulnerable components of moisture-dependent ecosystems under intensifying drought.

## 1. Introduction

Climate warming is intensifying drought across terrestrial ecosystems by altering precipitation patterns and increasing atmospheric evaporative demand, which together increase drought frequency, severity, and recovery time [1,2]. Rapid-onset drought events, or flash droughts, have sharpened this threat because these events involve the unusually rapid development of soil moisture deficits under combined precipitation deficits and high atmospheric evaporative demand, allowing them to suppress vegetation activity within a short period and leave ecological legacies after the meteorological anomaly has passed [3,4]. Most broad assessments of drought vulnerability, however, still center on seed plants, forests as tree-dominated systems, or agricultural vegetation, whereas non-seed plant lineages remain much less integrated into global syntheses despite their strong influence on water retention, carbon exchange, nutrient cycling, and microhabitat persistence [5,6]. This imbalance matters because drought does not act only through canopy dieback or productivity loss. It also propagates through understory and canopy microsites where plant groups with very different hydraulic strategies regulate moisture storage, nutrient retention, and the continuity of humid habitat [7,8,9]. Understanding drought sensitivity in these lineages is therefore necessary for a more comprehensive account of how forest and peatland systems adapt to a drying climate.

Ferns and mosses provide a particularly informative comparison because they are both ecologically consequential and physiologically distinct. Both groups are spore-producing non-seed plant lineages that often occupy moisture-dependent forests, peatlands, and epiphytic microsites; however, they differ strongly in vascular organization and hydration regulation [10]. Ferns are early diverging vascular plants that occupy terrestrial and epiphytic habitats across a wide climatic range and contribute substantially to diversity in humid forests, especially in montane and cloud forest systems where epiphytic assemblages are often highly developed [11,12]. Mosses, in turn, dominate many forest floors, peatlands, and canopy substrates, where they shape surface water retention, nutrient turnover, carbon storage, and fine-scale habitat structure [7,13]. The two lineages also differ fundamentally in how they acquire, store, and lose water. Ferns can rely on vascular transport, frond water storage, and, in many taxa, at least partial stomatal regulation, although the strength and evolutionary distribution of stomatal responsiveness remain heterogeneous across clades and developmental stages [14,15,16]. Mosses are more commonly poikilohydric and remain closely coupled to ambient moisture, depending more strongly on dehydration tolerance, metabolic downregulation, and rapid reactivation after rewetting [17,18]. These lineages offer a natural contrast for identifying which drought responses are broadly shared and which are rooted in fundamentally different hydraulic organization.

In ferns, drought responses are distributed across several interconnected physiological domains rather than expressed through a single dominant trait. Water deficit can reduce pigment content, gas exchange, and growth while altering fluorescence parameters that reflect the partitioning of absorbed energy between photochemistry and thermal dissipation [19,20]. At the same time, fern responses are strongly conditioned by variation in hydraulic design and stomatal behavior. Experimental studies indicate that active stomatal control operates in some lineages, whereas others show weak or inconsistent sensitivity to abscisic acid (ABA), which suggests that drought regulation in ferns is evolutionarily heterogeneous rather than physiologically uniform [16,21,22]. This heterogeneity becomes especially important in epiphytic taxa, where water supply depends less on buffered soil moisture and more on canopy humidity, interception, and short-term atmospheric inputs [23,24]. Recent syntheses therefore argue that fern drought biology is best interpreted through the joint influence of hydraulic traits, ecological habitat, and evolutionary history, rather than through direct analogy with angiosperm stress models [25,26].

Mosses present a different physiological framework. Because tissue hydration closely tracks environmental water availability, drought can shift rapidly from reduced photosynthetic activity to oxidative stress, membrane destabilization, and survival loss once protective capacity is exceeded [27,28,29]. At the same time, many mosses and other bryophytes retain deeply conserved stress response modules that are central to drought tolerance. Comparative and molecular evidence shows that abscisic acid signaling, chloroplast protection, antioxidant defense, and dehydration-responsive gene regulation are all prominent components of bryophyte stress biology, although their strength and coordination differ among taxa and habitats [17,30,31]. Moss drought responses are therefore not simply attenuated versions of vascular plant responses. Rather, they reflect a distinct balance among constitutive tolerance, reversible metabolic shutdown, and cumulative injury during prolonged drying [18,32]. That distinction is central here because an equivalent decline in water content (WC) can signify very different physiological states in a poikilohydric moss cushion and a vascular fern frond.

Despite substantial progress in lineage-specific studies, the available evidence remains difficult to generalize across taxa, habitats, and response domains. Many drought experiments focus on a single species, a single habitat, or a narrow set of traits, which makes it difficult to determine whether pigment decline, photochemical injury, osmotic adjustment, hormonal signaling, and growth suppression shift together or decouple under stress [33,34,35]. Cross-lineage comparison is especially limited, even though ferns and mosses often coexist in shaded forest understories, montane systems, and epiphytic assemblages that are increasingly exposed to warming-driven atmospheric drying [12,24]. Current understanding is also constrained by insufficient attention to ecological habitat and drought intensity. Epiphytic ferns experience strong coupling to canopy exposure and atmospheric humidity. In contrast, terrestrial ferns can draw on more buffered soil water pools, so the same nominal drought treatment may not represent the same physiological transition across habitats [23,36]. Likewise, mild, moderate, and severe drought need not map onto equivalent stages of stress progression in ferns and mosses because hydration loss, photoprotection, and oxidative injury can all shift nonlinearly with increasing stress intensity [25,28]. A comparative synthesis that integrates physiological domains, ecological habitats, and climatic context is therefore needed before broader ecological consequences can be interpreted with confidence.

To address this gap, we conducted a global meta-analysis of 3272 paired observations from experimental drought studies across multiple species, ecological habitats, and drought-intensity classes. We aimed to clarify: (1) whether ferns and mosses differ systematically in photosynthetic damage, oxidative stress, and hormone regulation under drought, and (2) whether ecological habitat and leaf functional traits explain variation in drought responses among ferns, which occupy habitats that differ markedly in water supply, substrate buffering, and exposure to atmospheric drying. We hypothesized that (i) drought would depress physiological performance in both groups, with stronger effects in mosses because of their greater dependence on external water availability, and (ii) fern drought sensitivity would be greater in ecological groups exposed to more intermittent water supply and lower substrate buffering, particularly epiphytic, canopy, tank-forming, and xerophytic taxa, and would vary with leaf traits associated with water retention and carbon gain.

## 2. Results

### 2.1. Drought Effects on Physiological Traits of Ferns and Mosses

**Figure 1:**
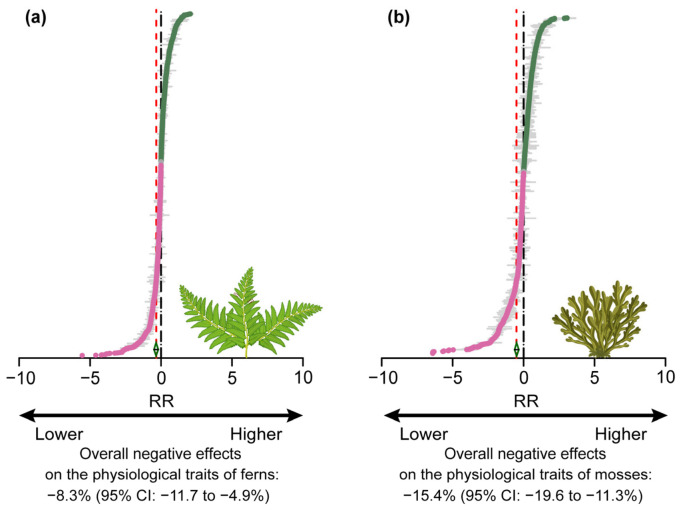
Overall effects of drought on the physiological traits of ferns (**a**) and mosses (**b**). Log response ratios (RR) indicate effect sizes. Dots show mean estimates, and grey lines show 95% confidence intervals (CIs). Green dots indicate significant positive effects, pink dots significant negative effects, and grey dots non-significant effects. The black dashed line marks RR = 0, while the overall effect size is represented by the red dashed line and the green diamond (with 95% CI in the black line).

**Figure 2:**
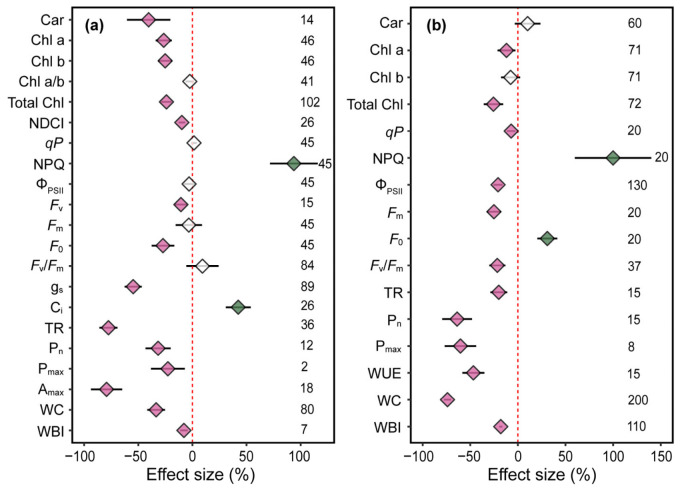
Drought effects on the key indicators of plant photosynthetic efficiency, pigment composition, and water relations of ferns (**a**) and mosses (**b**). Diamonds show mean effect sizes, and black lines show 95% confidence intervals. The red dashed line marks effect size = 0. Green symbols indicate significant positive effects, pink symbols indicate significant negative effects, and white symbols indicate nonsignificant effects. Numbers indicate independent observations. Car, carotenoid content; Chl a, chlorophyll a; Chl b, chlorophyll b; Chl a/b, chlorophyll a to b ratio; Total Chl, total chlorophyll; NDCI, normalized difference chlorophyll index; *qP*, photochemical quenching; NPQ, non-photochemical quenching; Φ_PSII_, effective quantum yield of photosystem II; *F*_v_, variable fluorescence; *F*_m_, maximal fluorescence; *F*_0_, minimum fluorescence; *F*_v_/*F*_m_, maximal quantum yield of photosystem II; g_s_, stomatal conductance; C_i_, intercellular carbon dioxide concentration; TR, transpiration rate; P_n_, net photosynthetic rate; P_max_, maximum photosynthetic rate; A_max_, maximum net photosynthetic rate; WC, water content; WBI, water band index; WUE, water use efficiency.

**Figure 3:**
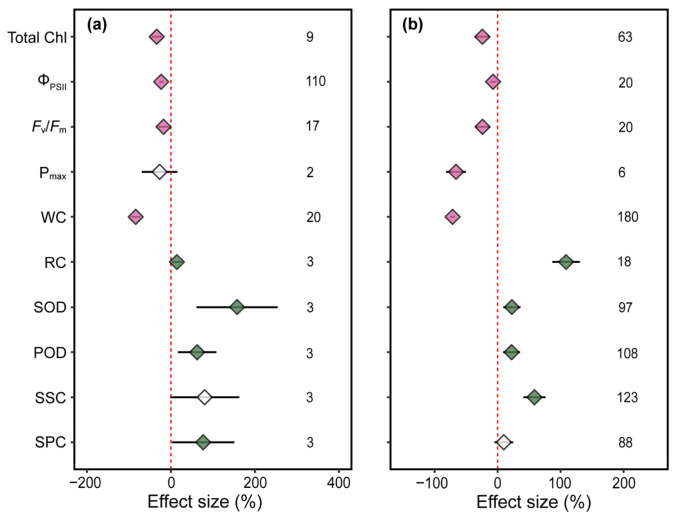
Effect size of drought on photosynthetic physiological traits and ecophysiological adaptive strategies in sphagnum (**a**) and non-sphagnum (**b**) mosses. Diamonds show mean effect sizes, and black lines show 95% confidence intervals. The red dashed line marks effect size = 0. Green symbols indicate significant positive effects, pink symbols indicate significant negative effects, and white symbols indicate nonsignificant effects. Numbers indicate independent observations. Total Chl, total chlorophyll; Φ_PSII_, effective quantum yield of photosystem II; *F*_v_/*F*_m_, maximal quantum yield of photosystem II; P_max_, maximum photosynthetic rate; WC, water content; RC, relative conductivity; SOD, superoxide dismutase; POD, peroxidase; SSC, soluble sugar; SPC, soluble protein.

Drought significantly impaired physiological performance in both lineages, with mosses exhibiting a greater overall decline than ferns. The mean overall decline was 8.3% in ferns (back-transformed 95% CI: 4.9–11.7%; Figure 1a; Table S1) compared with 15.4% in mosses (95% CI: 11.3–19.6%; Figure 1b; Table S1). Photosynthetic pigments and water-related traits responded differently between the two groups. In ferns, carotenoid content (Car) declined by 40.4%, chlorophyll a (Chl a) by 26.5%, chlorophyll b (Chl b) by 25.2%, total chlorophyll (Total Chl) by 24.0%, and normalized difference chlorophyll index (NDCI) by 9.7% (Figure 2a; Table S2). In mosses, chlorophyll a declined by 12.0% and total chlorophyll by 25.7%, whereas carotenoid content and chlorophyll b did not change significantly (Figure 2b; Table S3). When mosses were further categorized according to growth form into sphagnum and non-sphagnum groups, distinct response patterns emerged, with total chlorophyll being reduced by 33.8% in sphagnum (Figure 3a) and by 24.2% in non-sphagnum (Figure 3b). Fern fluorescence responses were characterized by decreases in variable fluorescence (*F*_v_) and minimum fluorescence (*F*_0_) together with a 93.8% increase in non-photochemical quenching (NPQ), whereas photochemical quenching (*qP*), effective quantum yield of photosystem II (Φ_PSII_), and maximum fluorescence (*F*_m_) showed no significant overall change (Figure 2a; Table S2). Mosses showed a broader decline in fluorescence performance. Photochemical quenching decreased by 7.1%, effective quantum yield by 20.8%, maximum fluorescence by 25.2%, and maximal quantum yield of photosystem II (*F*_v_/*F*_m_) by 21.6%, whereas non-photochemical quenching and minimum fluorescence increased by 99.9% and 30.9%, respectively (Figure 2b; Table S3). Notably, Φ_PSII_ exhibited a 23.1% reduction in sphagnum compared with a 7.2% decline in non-sphagnum, while the corresponding reductions in Fv/Fm were 17.6% and 23.9%, respectively (Figure 3a,b).

Gas exchange was strongly inhibited in both groups. In ferns, stomatal conductance (g_s_), transpiration rate (TR), net photosynthetic rate (P_n_), maximum photosynthetic rate (P_max_), and maximum net photosynthetic rate (A_max_) declined by 54.7%, 77.6%, 31.6%, 22.6%, and 79.3%, respectively. Water content and the water band index (WBI) declined by 33.5% and 7.8%, whereas intercellular carbon dioxide concentration (C_i_) increased by 42.4% (Figure 2a; Table S2). In mosses, transpiration rate, net photosynthetic rate, maximum photosynthetic rate, water use efficiency (WUE), water content, and the water band index declined by 20.1%, 63.8%, 60.3%, 46.7%, 73.9%, and 18.0%, respectively (Figure 2b; Table S3). Within the moss lineage, P_max_ remained unchanged in sphagnum but dropped sharply by 66.1% in non-sphagnum, and water content decreased by 83.8% in sphagnum and by 71.5% in non-sphagnum (Figure 3a,b).

**Figure 4:**
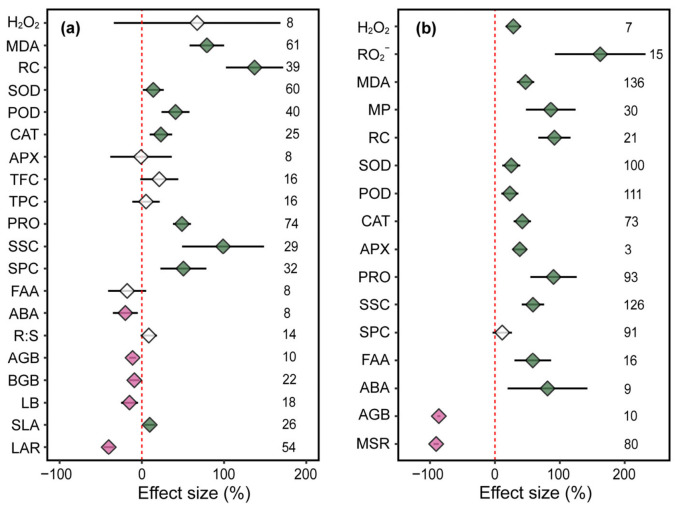
Drought effects on the osmoregulatory substances, hormone regulation, and growth strategy parameters of ferns (**a**) and mosses (**b**). Diamonds show mean effect sizes, and black lines show 95% confidence intervals. The red dashed line marks effect size = 0. Green symbols indicate significant positive effects, pink symbols indicate significant negative effects, and white symbols indicate nonsignificant effects. Numbers indicate independent observations. H_2_O_2_, hydrogen peroxide; MDA, malondialdehyde; RC, relative conductivity; SOD, superoxide dismutase; POD, peroxidase; CAT, catalase; APX, ascorbate peroxidase; TFC, total flavonoids; TPC, total phenols; PRO, proline; SSC, soluble sugar; SPC, soluble protein; FAA, free amino acids; ABA, abscisic acid; R:S, root to shoot ratio; AGB, aboveground biomass; BGB, belowground biomass; LB, leaf biomass; SLA, specific leaf area; LAR, leaf area ratio; RO_2_^−^, superoxide generation rate; MP, membrane permeability; MSR, mean survival rate.

Oxidative stress, osmotic regulation, hormone dynamics, and growth also differed between lineages. In ferns, malondialdehyde (MDA) and relative conductivity (RC) increased by 79.1% and 137.1%. Antioxidant enzymes were generally upregulated, with superoxide dismutase (SOD), peroxidase (POD), and catalase (CAT) increasing by 13.9%, 40.9%, and 23.1%, respectively (Figure 4a; Table S4). Proline (PRO), soluble sugar (SSC), and soluble protein (SPC) content increased by 48.8%, 98.8%, and 50.5%, respectively. Fern specific leaf area (SLA) increased by 9.6%, but aboveground biomass (AGB), belowground biomass (BGB), leaf biomass (LB), and leaf area ratio (LAR) declined by 11.3%, 9.1%, 15.0%, and 40.3%, respectively (Figure 4a; Table S4). Mosses showed stronger oxidative injury, with hydrogen peroxide (H_2_O_2_), superoxide generation rate (RO_2_^−^), malondialdehyde, membrane permeability (MP), and relative conductivity increasing by 28.4%, 162.2%, 47.2%, 86.0%, and 91.6%, respectively, while hydrogen peroxide did not change significantly in ferns. Moss antioxidant activity also increased, with superoxide dismutase, peroxidase, catalase, and ascorbate peroxidase (APX) rising by 25.2%, 23.0%, 42.1%, and 38.2%, respectively (Figure 4b; Table S5). Proline, soluble sugar, and free amino acids (FAA) increased by 90.2%, 58.6%, and 58.3%, respectively. Abscisic acid increased by 81.0% in mosses (Figure 4b; Table S5) but decreased by 20.1% in ferns (95% CI: 5.0–35.2%; Figure 4a; Table S4). Moss growth was strongly suppressed, with aboveground biomass declining by 86.4% and mean survival rate (MSR) by 90.6% (Figure 4b; Table S5). The differences between sphagnum and non-sphagnum also show up in how they handle oxidative stress and osmotic regulation, where relative conductivity increased by 13.8% in sphagnum and by 108.7% in non-sphagnum (Figure 3a,b). SOD activity rose by 157.6% and 22.6%, respectively, and POD activity increased by 62.4% and 22.1%, respectively. For osmotic regulators, soluble sugar content showed no significant change in sphagnum but increased by 58.5% in non-sphagnum, whereas soluble protein content was elevated by 76.8% in sphagnum but remained unchanged in non-sphagnum (Figure 3a,b).

### 2.2. Drought Effects on Epiphytic Ferns Differing in Ecological Habitat

**Figure 5:**
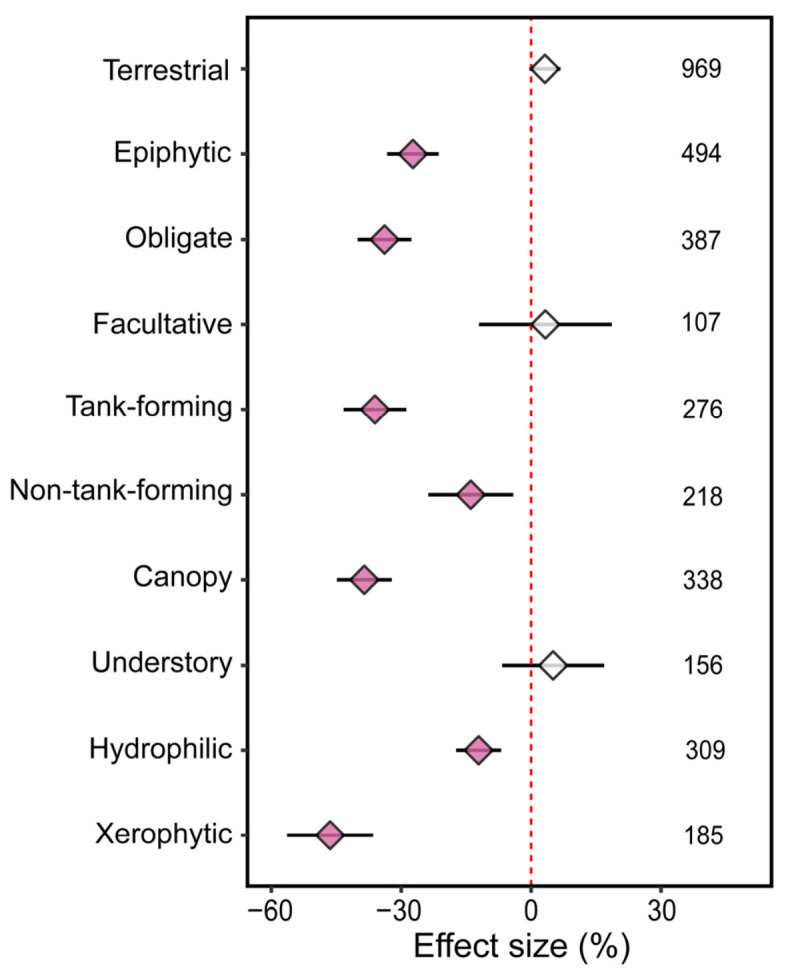
Drought responses of epiphytic ferns across ecological habitats. Diamonds show mean effect sizes, and black lines show 95% confidence intervals. The red dashed line marks effect size = 0. Pink symbols indicate significant negative effects and white symbols indicate nonsignificant effects. Numbers indicate independent observations.

Fern responses varied strongly with ecological habitat. Terrestrial ferns showed no significant overall change, whereas epiphytic ferns declined by 27.3% under drought (Figure 5; Table S6). Among epiphytes, obligate epiphytes declined by 33.8%, while facultative epiphytes showed no significant response. Tank-forming epiphytes and non-tank-forming epiphytes declined by 36.1% and 13.9%, respectively. Canopy epiphytes declined by 38.5%, whereas understory epiphytes did not differ significantly from controls. Hygrophytic epiphytes declined by 12.1%, and xerophytic epiphytes declined by 46.4%, which was the strongest response among the ecological groups analyzed (Figure 5; Table S6).

### 2.3. Response Patterns of Physiological Traits Across Experimental Drought Intensity

Responses across the experimental drought intensity gradient were clearer when physiological traits were grouped into functional categories (Figures S1 and S2; Tables S7–S12). In ferns, low drought mainly affected water status and early biochemical stress indicators, with water content decreasing by 18.2%, malondialdehyde increasing by 48.2%, and proline and soluble protein increasing by 26.6% and 31.8%, respectively. Under moderate drought, the response expanded across four domains. Pigment traits declined, with carotenoids, chlorophyll a, chlorophyll b, and total chlorophyll decreasing by 44.5%, 22.3%, 21.6%, and 21.3%, respectively. Water status deteriorated further, with water content declining by 30.3%. Oxidative stress and antioxidant defense intensified, as malondialdehyde increased by 81.7% and peroxidase and catalase increased by 30.7% and 21.3%, respectively. Osmotic adjustment also strengthened, with proline, soluble sugar, and soluble protein increasing by 36.6%, 174.0%, and 68.2%, respectively. Under severe drought, the same response domains showed stronger shifts, including pigment reductions of 33.7% to 43.9%, a 41.1% decline in water content, a 108.1% increase in malondialdehyde, increases in peroxidase and catalase of 97.4% and 64.6%, and increases in proline, soluble sugar, and soluble protein of 88.0%, 404.2%, and 59.1%, respectively (Figure S1; Tables S7–S9).

In mosses, the response gradient was steeper and remained centered on three dominant domains: hydration loss, oxidative stress, and osmotic adjustment. Under low drought, water content decreased by 52.7%, while total chlorophyll increased by 16.6%, and malondialdehyde and catalase increased by 31.7% and 19.9%, respectively; the increase in total chlorophyll was interpreted cautiously as a possible early-stage compensatory response rather than evidence of a beneficial drought effect. Under moderate drought, pigment decline became more evident, with chlorophyll a and total chlorophyll decreasing by 11.3% and 18.3%, while water content declined by 67.2%. At the same drought level, antioxidant and osmotic responses strengthened, with peroxidase and catalase increasing by 35.7% and 67.8%, malondialdehyde increasing by 27.7%, and proline and soluble sugar increasing by 92.6% and 65.7%, respectively. Under severe drought, hydration loss and biochemical disruption intensified further, with chlorophyll b and total chlorophyll decreasing by 19.7% and 45.1%, water content declining by 82.0%, malondialdehyde increasing by 71.0%, peroxidase and catalase increasing by 20.4% and 34.4%, and proline and soluble sugar increasing by 135.2% and 81.3%, respectively (Figure S2; Tables S10–S12).

### 2.4. Climate and Trait Moderators of Fern Drought Responses

**Figure 6:**
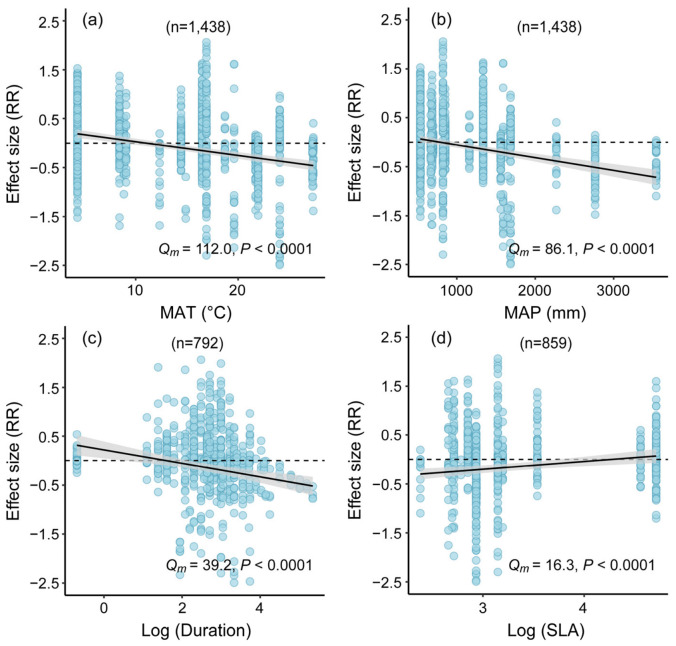
Mean annual temperature ((**a**), MAT), mean annual precipitation ((**b**), MAP), drought duration (**c**), and specific leaf area ((**d**), SLA) modulate fern physiological responses to drought. The black dashed line indicates null effects. Bubbles show independent observations. Black lines show predicted mean effect size, and light grey bands show 95% confidence intervals.

Meta-regression showed that the climate of provenance and leaf functional traits together mediated the responses of fern physiological traits to drought. Specifically, drought-induced changes in physiological traits were significantly influenced by mean annual temperature (MAT) of provenance (*Q_m_* = 112.0, *p* < 0.0001, Figure 6a), mean annual precipitation (MAP) of provenance (*Q_m_* = 86.1, *p* < 0.0001, Figure 6b), drought duration (*Q_m_* = 39.2, *p* < 0.0001, Figure 6c) and specific leaf area (*Q_m_* = 16.3, *p* < 0.0001, Figure 6d). Together, these moderators explained a substantial portion of the variation in physiological responses to drought, with a high combined explanatory power (total *Q_m_* = 253.6, *p* < 0.0001).

Notably, the response of fern physiological traits to drought gradually weakened as the mean annual temperature of the provenance increased. When this value was below approximately 11 °C, drought had a positive effect on the physiological traits of ferns; however, when the mean annual temperature of the provenance exceeded 11 °C, the drought effect turned from positive to negative, exhibiting a negative effect (Figure 6a). A similar threshold pattern was observed along the precipitation gradient. When the mean annual precipitation of the provenance was below 690 mm, drought had a positive effect on physiological traits, whereas above 690 mm, the effect became negative (Figure 6b). Meanwhile, the magnitude of fern physiological responses decreased as drought duration increased (Figure 6c). In addition, specific leaf area was positively associated with RR, indicating that drought effects became less negative as SLA increased; thus, higher-SLA fern species tended to show attenuated declines across the measured physiological traits (Figure 6d).

## 3. Discussion

### 3.1. Divergent Trajectories of Photosynthetic Regulation and Photochemical Injury

This synthesis shows that drought suppresses physiological performance in both ferns and mosses, but the pathways of decline differ in ways that are consistent with major differences in anatomy and hydration strategy [20,25]. Ferns retained relatively stable pooled *F*_v_/*F*_m_ while showing strong reductions in pigments and carbon assimilation, along with a major increase in nonphotochemical quenching [33,37]. This pattern suggests that many fern species preserved core photochemical function through enhanced energy dissipation even when carbon gain and water status declined [20]. Mosses showed a more severe syndrome, combining large decreases in water content and photosynthetic rates with coordinated deterioration in *F*_m_, Φ_PSII_, and *F*_v_/*F*_m_ and an increase in *F*_0_. That combination is more consistent with direct damage to photosystem II or limited capacity to prevent photochemical disruption once tissues dehydrate [27,38]. The contrasting responses of P_max_ and A_max_ further indicate that drought affected carbon gain more strongly than the intrinsic photosynthetic capacity of ferns. P_max_ primarily reflects the maximum biochemical capacity of photosynthetic carbon fixation under saturating light. In contrast, A_max_ integrates additional limitations imposed by stomatal regulation, carbon dioxide (CO_2_) diffusion, respiratory carbon loss, and metabolic constraints. Therefore, the stronger decline in A_max_ suggests that drought-induced stomatal closure and reduced CO_2_ availability constrained net carbon assimilation, even though the photosynthetic apparatus retained relatively stable photochemical function. This pattern is consistent with the increased NPQ and stable *F*_v_/*F*_m_ observed in ferns, indicating that photochemical energy dissipation may have protected PSII while carbon assimilation was downregulated under drought stress.

These lineage differences agree with recent mechanistic syntheses of plant water stress and drought biology [35,39]. Drought responses are not limited to one pathway, but emerge from coordinated changes in water transport, photoprotection, redox control, and metabolic adjustment [5]. In seed-free plants, the balance among these processes is shaped strongly by whether tissues maintain internal hydraulic continuity or track environmental water content more directly [40,41]. Fern sporophytes often possess enough vascular and stomatal control to delay severe photochemical damage during moderate drought, although this buffering clearly fails in some humidity-dependent lineages [26,42]. Mosses, by contrast, frequently depend on external water films and rapid tissue rehydration [27,43]. Once dehydration exceeds the recovery capacity of the species or life stage, fluorescence declines can become abrupt, and oxidative stress can intensify [44,45]. Recent work on moss drought tolerance also emphasizes that tolerance is unevenly distributed, with large differences among species in the coordination of ABA signaling, protective proteins, antioxidant metabolism, and recovery kinetics [31,32].

### 3.2. Oxidative Stress Coordination, Abscisic Acid Divergence, and Growth Consequences

Both ferns and mosses increased antioxidant and osmotic responses under drought, but stronger defense activation did not necessarily indicate more effective physiological protection. In ferns, increases in superoxide dismutase, peroxidase, catalase, proline, soluble sugar, and soluble protein coincided with strong declines in gas exchange and higher membrane damage indicators [33,46]. In mosses, antioxidant activation was even stronger, but so were hydrogen peroxide accumulation, membrane permeability, and survival loss. The most parsimonious interpretation is that defense activation in mosses often reflects more intense stress exposure rather than more effective protection [29,47]. In other words, the antioxidant system responded, but the response was often insufficient relative to the scale of dehydration and photochemical disruption [32,48].

The divergent abscisic acid responses further suggest different drought-regulation strategies in mosses and ferns. Mosses showed a marked ABA increase under drought, consistent with evidence that dehydration responses in bryophytes are often associated with ABA accumulation and downstream protective pathways [31,49]. In crop and other seed-plant models, drought responses are often framed around ABA accumulation and downstream regulatory networks that coordinate stomatal regulation, osmotic adjustment, antioxidant defense, and stress-responsive gene expression [50]. Recent maize evidence further illustrates this canonical seed-plant framework, showing that the guard-cell-expressed transcription factor ZmMYB56 promotes stomatal closure and drought tolerance through transcriptional regulation of ZmTOM7 [51]. By contrast, ferns showed a negative pooled ABA response, indicating that ABA-mediated water-stress regulation in seed-free vascular plants does not necessarily follow the canonical seed-plant pattern [52,53]. Several non-mutually exclusive explanations may account for this pattern. First, fern ABA responses may be transient, and measurements taken after the initial stress phase could capture later ABA catabolism, conjugation, or depletion rather than the early ABA peak [52]. Second, differences among source studies in whether free or total ABA was measured may have contributed to variation in the pooled estimate. Third, because stomatal and hydraulic regulation in ferns is heterogeneous across lineages, ABA accumulation may not be the dominant drought-regulatory signal in all fern species [16]. Therefore, the negative pooled ABA response should be interpreted cautiously as a hypothesis-generating pattern rather than evidence that ferns lack ABA-related drought regulation. Experimental work has shown weak or absent stomatal responses to ABA in some fern and lycophyte species, while other studies indicate partial conservation or lineage-specific convergence in the underlying signaling toolkit [15,21,52]. Our meta-analysis therefore supports a more constrained interpretation: ABA increased in mosses but decreased in ferns under drought, suggesting that ABA-related drought responses are not uniform across these non-seed plant lineages and that fern responses should not be interpreted through the canonical seed-plant ABA model alone. This divergence in ABA response may partly contribute to the contrasting physiological profiles observed between mosses and ferns.

### 3.3. Ecological Habitat, Drought Thresholds, and Climatic Modulation of Fern Sensitivity

The strongest fern responses occurred in epiphytes, especially obligate, canopy, tank-forming, and xerophytic groups. This result indicates that drought sensitivity in ferns is not determined only by phylogenetic position, but also by ecological specialization [54,55]. Epiphytes operate with intermittent water supply, shallow storage pools, and strong dependence on atmospheric conditions [56,57]. These constraints become especially acute in canopies where radiation load and vapor pressure deficit are high [58]. Recent studies from tropical cloud forests show that epiphytic ferns and orchids shift water source use under drought and depend strongly on fog and atmospheric moisture, while broader reviews emphasize that vascular epiphytes are among the plant groups most exposed to climate and habitat change [36,59,60]. Our results extend those insights by showing that ecological syndromes within epiphytic ferns translate into predictable differences in drought response magnitude.

The strong response of tank-forming epiphytes is also notable. Water impounding rosettes can buffer short dry spells when rainfall is frequent, but these same structures may provide limited protection during prolonged drought if storage is not replenished [24,61]. Xerophytic epiphytes also showed larger physiological responses than hygrophytic taxa in the pooled analysis. This pattern likely reflects habitat edge specialization and strong trait responsiveness under intermittent water supply, rather than necessarily indicating lower survival tolerance [54,62]. Under stronger experimental drought treatments, quantified as reductions in water supply or substrate/soil moisture relative to controls, these taxa may show large shifts in the measured physiological traits because these traits may respond actively to water deficit. Thus, in this synthesis, larger trait responses should be interpreted as physiological responsiveness during stress, not as direct evidence of lower ecological tolerance or survival probability. Provenance climate effects support the broader interpretation that long-term climatic history shapes the physiological baseline from which drought responses unfold, as fern responses varied with MAT and MAP [63,64,65].

Notably, the effects of drought on the physiological traits of ferns exhibited clear climatic thresholds. Drought predominantly exerted positive effects when the MAT of the provenance was below 11 °C or the MAP was below 690 mm (Figure 6a,b). However, once these thresholds were exceeded, the drought response shifted from positive to negative. This pattern suggests that ferns originating from relatively cooler or drier habitats may be preadapted to water deficits and can even experience physiological enhancement under moderate drought conditions [55]. In contrast, taxa from warm and humid regions, having evolved under conditions of high-water availability, may possess physiological mechanisms that render them more sensitive to water scarcity [66,67]. When exposed to drought stress, these taxa are more likely to rapidly approach or surpass their physiological tolerance limits [40]. This also helps explain why hygrophilous groups often exhibit greater response magnitudes in experimental settings [68,69]. Their native climatic backgrounds place them closer to the upper boundary of their water requirements, making any additional water deficit more likely to trigger pronounced physiological responses.

SLA, a key leaf functional trait, positively moderated fern physiological responses to drought. Because drought effects became less negative as SLA increased, this pattern should be interpreted as attenuated drought-induced declines in higher-SLA ferns rather than as greater response magnitude or stronger resilience. One plausible explanation is that high-SLA ferns, which typically have thinner leaves, lower construction costs, and faster resource turnover, may maintain short-term physiological adjustment more effectively under experimental water limitation [70,71]. Such adjustment may involve faster resource allocation to osmotic regulation, photoprotection, or recovery of carbon gain after mild to moderate stress. By contrast, low-SLA species are generally associated with more conservative resource-use strategies, including greater structural investment and slower physiological turnover, which may limit rapid adjustment when water availability declines [72]. However, the positive association between SLA and drought response should not be interpreted as evidence that high-SLA species are universally more drought-tolerant. Fast resource-acquisition strategies may confer advantages under short-term or moderate stress, but they can become less advantageous under prolonged or severe dehydration because they are often associated with lower structural protection and potentially greater exposure to water loss [73]. Overall, the observed SLA pattern is consistent with a trade-off between rapid physiological responsiveness and structural protection, providing a plausible explanation for variation in drought-response patterns among fern species with contrasting leaf functional strategies.

### 3.4. Limitations and Future Implications

Several common limitations should be recognized, although they do not weaken the central inference of this synthesis. Studies differed in how drought was imposed, particularly in the relative contributions of substrate or soil water deficit, atmospheric drying, and stress duration, which is difficult to control when comparing epiphytic, terrestrial, and peatland systems. For epiphytic species, the external treatment classes used here should therefore not be interpreted as direct estimates of internal plant water status. Recovery after rewetting was also reported less consistently than decline during dehydration, so the present dataset resolves the stress phase more clearly than the resilience phase. This issue is particularly relevant for mosses because some photosynthetic and fluorescence traits may reflect the hydration state at measurement or post-drought rehydration capacity rather than only the cumulative effect of drought exposure. Thus, the magnitude of physiological response should be interpreted as trait responsiveness during stress, rather than as a direct measure of survival-based drought tolerance. Taxonomic and life stage coverage remains uneven as well, particularly for moss hormonal traits and several specialized epiphytic fern guilds. Some traits routinely measured in fern leaves are difficult to obtain or are not directly comparable in mosses, contributing to uneven trait coverage between the two lineages. Moreover, for mosses, insufficient species-level ecological classification in the source studies precluded the subgroup analyses by ecological habitat that were conducted for ferns. Likewise, the geographic distribution of fern and moss observations is uneven across continents, a pattern inherent in the available studies and that limits truly paired, site-matched comparisons. Accordingly, our cross-lineage comparison should be interpreted as a synthesis of the fern and moss species represented in the available experimental literature, rather than as a complete representation of the full global diversity of either lineage. Thus, the pooled estimates should be viewed as broad central tendencies within the available dataset, not as universal drought-response traits of all ferns or all mosses. These constraints are typical of an emerging comparative literature and mainly define the next level of resolution that the field now needs. One priority is trajectory-based experimentation that follows dehydration and rehydration together while measuring fluorescence, water status, oxidative balance, osmolytes, and abscisic acid within the same individuals, because that design can locate the transition from reversible regulation to irreversible injury more precisely. A second priority is to embed physiology in real canopy and peatland microclimates, where atmospheric dryness, cloud immersion, and short-lived water inputs can govern exposure more directly than precipitation totals alone. Integrating those measurements with trait spectra, phylogenetic structure, and habitat-resolved monitoring should make it possible to identify lineage-specific drought thresholds and to forecast which assemblages are most likely to lose function first as drying intensifies. That shift from trait responses to threshold-based prediction would substantially strengthen forecasts of how humidity-dependent vegetation reshapes carbon storage, moisture retention, and biodiversity support in forests and peatlands under continued climate change.

## 4. Materials and Methods

### 4.1. Data Collection

To systematically assess drought impacts on the physiological traits and regulatory mechanisms of ferns and mosses, we compiled a global dataset. Through extensive searches of the China National Knowledge Infrastructure (CNKI), Google Scholar, and Web of Science, we identified relevant studies covering all peer-reviewed literature in these databases from 1 January 1980 to 31 December 2025 (Figure S3). The search employed the following combination of keywords: (drought* OR dry OR reduced precipitation OR decreased rainfall OR reduced soil moisture OR decreased water OR drying OR rain shelter OR rain cover OR water stress OR water deficit) AND (ferns* OR fern plants OR bryophyte OR sphagnum OR moss* OR moss plants OR pteridophyte) AND (physiological traits* OR photosynthetic pigments OR chlorophyll OR carotenoids OR chlorophyll fluorescence OR photochemical quenching OR non-photochemical quenching OR gas exchange OR photosynthetic rate OR transpiration rate OR stomatal conductance OR antioxidant enzymes OR superoxide dismutase OR peroxidase OR catalase OR malondialdehyde OR proline OR soluble sugar OR soluble protein OR abscisic acid OR biomass OR specific leaf area).

Eligible studies were further screened according to the following criteria: (1) environmental conditions were identical between control and drought treatments except for water supply or substrate water availability, while studies along natural precipitation gradients were excluded; (2) only studies on ferns and mosses reporting quantifiable physiological traits were included; (3) data were limited to control and drought treatments. Studies that incorporated additional global change factors, such as warming or elevated carbon dioxide, were excluded; (4) studies involving heavy metals, radiation, hormones, or herbicide application were omitted, as these factors may confound drought responses; (5) for each treatment group, the mean, standard deviations (s.d.), and replication counts (n) must be explicitly reported or derivable. In cases where a study encompassed multiple species or multiple experimental sites, each species or each experimental site was treated as an independent case in the analysis. For studies meeting the inclusion criteria, we extracted the mean, s.d., and sample size of photosynthetic pigment indicators, chlorophyll fluorescence parameters, gas exchange parameters, water status indicators, oxidative stress and antioxidant enzyme indicators, osmotic adjustment substances, hormone contents, as well as growth and resource allocation indicators from the main text, tables, and Supplementary Materials for both the control group and the drought treatment group. For gas exchange traits, P_max_ was defined as the maximum photosynthetic capacity measured under saturating light conditions, representing the potential capacity of photosynthetic carbon fixation. A_max_ was defined as the maximum net CO_2_ assimilation rate obtained from gas-exchange measurements, reflecting the realized carbon gain after considering respiratory losses and diffusional limitations. For mosses, these drought-treatment values were extracted according to the measurement status reported in the original studies; when photosynthetic or fluorescence traits were measured after standardized rehydration, they were interpreted as hydrated physiological capacity following drought exposure rather than as instantaneous performance during ongoing dehydration. The graphical data were digitized using GetData software (version 2.26; http://www.getdata-graph-digitizer.com/, accessed on 15 September 2025).

For each study, we recorded geographic coordinates, including longitude, latitude, and elevation, together with climatic variables, namely MAT and MAP. We also compiled study-specific information, including species identity and ecological habitat. Ecological habitat was classified as terrestrial or epiphytic, and epiphytic species were further categorized as obligate or facultative, tank-forming or non-tank-forming, canopy or understory, and hygrophytic or xerophytic. We additionally recorded drought intensity (low, <10% reduction in watering/simulated precipitation or <20% decline in substrate or soil moisture; moderate, 10–50% reduction in watering/simulated precipitation or 20–50% decline in substrate or soil moisture; severe, >50% reduction in watering/simulated precipitation or >50% decline in substrate or soil moisture). These thresholds describe external treatment intensity. For epiphytic species, “soil moisture” was not treated as literal soil-water stress; when original studies used epiphytic or artificial substrates, intensity was coded based on reductions in watering, misting, simulated precipitation, or moisture in the reported growth substrate. Plant water-status variables, when available, were extracted as response traits rather than as the basis for defining intensity, because they were too inconsistently reported to provide a common classification across all studies. Because drought treatments differed among the source studies, these intensity classes should be regarded as approximate operational categories based on reported treatment information rather than direct measures of physiological dehydration, particularly for epiphytic ferns and poikilohydric mosses. Throughout the analysis, “drought” refers to experimentally imposed water-deficit treatments as reported by the original studies, whereas “desiccation tolerance” refers specifically to survival and recovery after severe tissue dehydration. According to the corresponding coordinates, the missing climate data for field studies were obtained from the WorldClim database. It is important to note that although all drought treatments in this study were conducted under controlled laboratory conditions, the geographic coordinates (longitude, latitude, and elevation) and the corresponding climatic data (MAT and MAP) included in our analyses represent the original habitat information of each plant species at their collection sites. These variables were incorporated as moderators in our models to examine how a species’ native climatic background influences its physiological responses to subsequent laboratory-simulated drought conditions.

**Figure 7:**
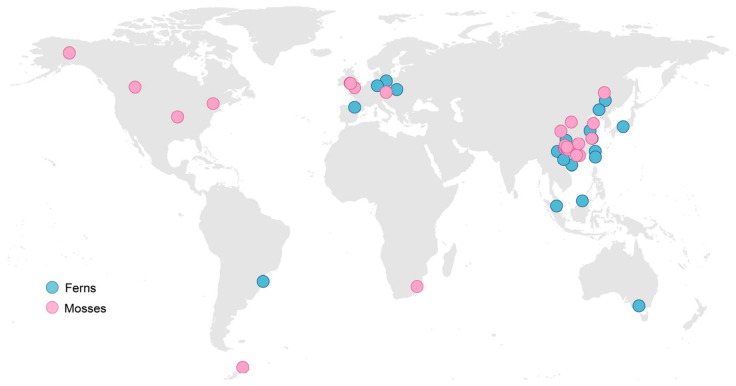
Global distribution of studies on the physiological traits of ferns and mosses under drought included in this meta-analysis. Blue points denote fern functional types, and pink points denote mosses.

We retrieved specific leaf area (mm^2^ mg^−1^) from the TRY plant trait database [74] for each fern, adopting this commonly used leaf functional trait as our continuous predictor. After removing duplicates and outliers beyond three standard deviations from the species mean, SLA values were averaged for each species. Ultimately, we constructed a dataset consisting of 3272 individual effect size data points, with each data point representing a pairwise comparison of a particular physiological trait of a single species under drought versus control conditions. These data points were derived from 35 species of ferns and 41 species of mosses in 46 studies (Figure 7 and Dataset S1). The fern dataset consisted entirely of leptosporangiate, true, megaphyllous ferns, so broad taxonomic contrasts such as eusporangiate versus leptosporangiate ferns, lycophytes versus true ferns, or microphyllous versus megaphyllous groups could not be analyzed. Therefore, fern subgroup analyses focused on ecological habitat. The moss dataset included both sphagnum and non-sphagnum mosses; we therefore added a subgroup analysis by moss type, using this ecologically meaningful distinction. For ferns, the extracted data primarily represented sporophyte fronds because fern gametophytes were rarely examined in the eligible studies. For mosses, the extracted measurements generally represented the dominant gametophyte stage.

### 4.2. Data Analysis

This meta-analysis was performed in accordance with the PRISMA guidelines. To quantify the effects of drought on the physiological traits of ferns and mosses, we used the natural log-transformed response ratio (RR) as the metric for effect size [75]. RR was calculated as follows:

(1) $$RR=ln\left(\frac{{\bar{X}}_{t}}{{\bar{X}}_{c}}\right)$$

where ${\bar{X}}_{t}$ and ${\bar{X}}_{c}$ denote the means for drought and control conditions, respectively. The associated variance (*v*) of RR was derived as:

(2) $$v=\frac{S_{t}^{2}}{N_{t}{\bar{X}}_{t}^{2}}+\frac{S_{c}^{2}}{N_{c}{\bar{X}}_{c}^{2}}$$

Here, $S_{t}$ and $S_{c}$ are the s.d., $N_{t}$ and $N_{c}$ are the sample sizes for the drought and control groups, respectively.

Weights for individual response ratios were determined as follows:

(3) $$w=\frac{1}{v+\tau^{2}}$$

where $\tau^{2}$ is the between-study variance common to all comparisons, calculated as:

(4) $$\tau^{2}=\frac{\sum_{i=1}^{m}w_{i}{{(RR}_{i}-\bar{RR})}^{2}-(m-1)}{\sum_{i=1}^{m}w_{i}-\frac{\sum_{i=1}^{m}w_{i}^{2}}{\sum_{i=1}^{m}w_{i}}}$$

with *m* being the total number of experiments and $w_{i}$ the weight for the *i*th experiment in the meta-analysis.

The weighted overall effects ($\bar{RR}$) were calculated as:

(5) $$\bar{RR}=\frac{\sum_{i=1}^{m}w_{i}\times {(RR}_{i})}{\sum_{i=1}^{m}w_{i}}$$

The percent response of physiological traits of ferns and mosses to drought was calculated as:

Percentage (%) = 100 × [exp (RR) − 1](6)

We assessed the impact of drought on the physiological traits of ferns and mosses by conducting a multilevel mixed-effects meta-analysis using the ‘rma.mv’ function in the ‘metafor’ package [76]. To account for non-independence among effect sizes and heterogeneity arising from study-specific conditions, we included study identity (ID) as a random effect in all models. Statistical significance of drought effects was inferred when the 95% confidence interval (CI) around the mean effect size did not include zero (*p* < 0.05) [77]. We assessed heterogeneity in effect sizes using Cochran’s *Q*-test, which evaluates whether the observed variation exceeds that expected by sampling error alone [78,79]. The analysis revealed substantial residual heterogeneity in drought responses for both ferns (*Q*_t_ = 670,032.4, *p* < 0.0001) and mosses (*Q*_t_ = 2,425,846.3, *p* < 0.0001; see Table S13), justifying subsequent exploration of moderator variables to explain this variation.

We conducted a univariate mixed-effects meta-regression analysis to examine how the effects of drought on physiological trait variation in ferns and mosses are influenced by climate of provenance (MAT and MAP), leaf functional traits (SLA), and drought duration. To assess the significance of moderator effects, we used the heterogeneity statistic (*Q_m_*) and its associated *p* value, with *p* < 0.05 considered evidence that the moderator significantly affects variation in effect size. The restricted maximum likelihood approach was used to obtain parameter estimates [80].

We tested for potential publication bias using multiple statistical approaches, including funnel plot inspection, Egger’s regression test [81], and Rosenberg’s fail-safe numbers [82], thereby evaluating the reliability of the study findings. The funnel plots of the fern and moss datasets both showed slight asymmetry (Figure S4), and the results of Egger’s test were significant (ferns: *z* = −6.14, *p* < 0.0001; mosses: *z* = −2.33, *p* = 0.0199). Nevertheless, fail-safe analysis revealed that 2.80 × 10^4^ and 3.14 × 10^7^ additional null result studies would be required to raise the combined *p* value of the fern and moss datasets to 0.05, respectively. Therefore, we consider that publication bias does not compromise the interpretation of our findings [83]. All statistical analyses were performed using R v.4.5.1 [84].

## 5. Conclusions

This global meta-analysis shows that drought suppresses physiological performance in both ferns and mosses, but the sequence and severity of responses differ markedly between the two lineages. Ferns generally maintained greater photochemical stability while showing large declines in water status, gas exchange, and growth, together with strong increases in non-photochemical quenching and antioxidant activity. Mosses showed a steeper shift toward direct impairment of photosystem II, more serious oxidative damage, a large decline in mean survival rate, and a pronounced increase in abscisic acid. These contrasts indicate that ferns and mosses do not occupy a single drought-response continuum. Instead, they differ in the point at which stress shifts from regulation to injury and in the mechanisms that dominate that shift. Within ferns, ecological specialization strongly shaped drought sensitivity, with epiphytic taxa, especially obligate, canopy, tank-forming, and xerophytic groups, showing the largest declines. Mean annual temperature and precipitation of provenance, drought duration, and specific leaf area further modified fern responses, emphasizing that climatic background, exposure duration, and leaf functional strategy are integral to predicting fern drought sensitivity. Together, these findings identify ferns and mosses as distinct but complementary indicators of climatic drying and provide a quantitative basis for anticipating changes in moisture-dependent forest and peatland systems.

## Data Availability

The data that support the findings of this study are openly available in figshare at https://doi.org/10.6084/m9.figshare.31813693.

## Supplementary Materials

The following supporting information can be downloaded at: https://www.mdpi.com/article/10.3390/plants15142143/s1, Figure S1: Effect size of drought on physiological traits under low (a), moderate (b), and severe (c) drought intensities in ferns; Figure S2: Effect size of drought on physiological traits under low (a), moderate (b), and severe (c) drought intensities in mosses; Figure S3: Preferred reporting items for systematic reviews and meta-analyses (PRISMA) flowchart illustrating the study selection process for this meta-analysis; Figure S4: Funnel plots showing the relationship between meta-analytic residuals and standard error are used to detect funnel plot asymmetry for ferns dataset (a) and mosses dataset (b); Table S1: Summary of the results of the meta-analysis on the response of drought on the physiological traits of ferns and mosses at a global scale, using the response metric *RR* (%); Table S2: Summary of the results of the meta-analysis on the response of drought to the key indicators of plant photosynthetic efficiency, pigment composition, and water relations of ferns at a global scale, using the response metric *RR* (%); Table S3: Summary of the results of the meta-analysis on the response of drought to the key indicators of plant photosynthetic efficiency, pigment composition, and water relations of mosses at a global scale, using the response metric *RR* (%); Table S4: Summary of the results of the meta-analysis on the response of drought to the osmoregulatory substances, hormone regulation, and growth strategy parameters of ferns at a global scale, using the response metric *RR* (%); Table S5: Summary of the results of the meta-analysis on the response of drought to the osmoregulatory substances, hormone regulation, and growth strategy parameters of mosses at a global scale, using the response metric *RR* (%); Table S6: Summary of the results of the meta-analysis on the response of drought to epiphytic ferns across ecological habitats at a global scale, using the response metric *RR* (%); Table S7: Summary of the results of the meta-analysis on the response of drought under low drought intensities in ferns at a global scale, using the response metric *RR* (%); Table S8: Summary of the results of the meta-analysis on the response of drought under moderate drought intensities in ferns at a global scale, using the response metric *RR* (%); Table S9: Summary of the results of the meta-analysis on the response of drought under severe drought intensities in ferns at a global scale, using the response metric *RR* (%); Table S10: Summary of the results of the meta-analysis on the response of drought under low drought intensities in mosses at a global scale, using the response metric *RR* (%); Table S11: Summary of the results of the meta-analysis on the response of drought under moderate drought intensities in mosses at a global scale, using the response metric *RR* (%); Table S12: Summary of the results of the meta-analysis on the response of drought under severe drought intensities in mosses at a global scale, using the response metric *RR* (%); Table S13: Summary of global meta-analysis of the effects of drought on the physiological traits in ferns and mosses. Dataset S1: Sources of studies included in the meta-analysis on physiological traits of ferns and mosses under drought.
