# Supplementary material for: Contrasting Photochemical Stability and Oxidative Injury Shape Drought Responses in Ferns and Mosses
Source: Plants (Basel). 2026 Jul 11;15(14):2143. doi: 10.3390/plants15142143 (PMC13415622; doi:10.3390/plants15142143)
Supplement: Supplementary file 1 [file plants-15-02143-s001.zip › plants-4407180-supplementary-proofed.pdf]

Supplementary Information for

Contrasting photochemical stability and oxidative injury shape drought responses in ferns and mosses

Hui Zhang [1,2], Changhui Peng [3,4], Jiahuan Guo [2], Qiuyu Liu [5], Douglass F. Jacobs [6,7], Mei Yang [1], Mengke Huang [1], Huili Feng [1,*]

[1] Key Laboratory of Ministry of Education for Genetics and Germplasm Innovation of Tropical Special Trees and Ornamental Plants, School of Tropical Agriculture and Forestry (School of Agricultural and Rural Affairs, School of Rural Revitalization), Hainan University, Danzhou 571737, China; huizhang@hainanu.edu.cn (H.Z.); ym198214@hainanu.edu.cn (M.Y.); huangmengke@hainanu.edu.cn (M.H.); fenghuili@hainanu.edu.cn (H.F.)
[2] Hainan Baoting Tropical Forest Ecosystem Observation and Research Station, School of Ecology, Hainan University, Haikou 570228, China; guojiahuan@hainanu.edu.cn (J.G.)
[3] Department of Biological Sciences, University of Quebec at Montreal, Montreal, Quebec H3C 3P8, Canada; peng.changhui@uqam.ca (C.P.)
[4] College of Geographic Science, Hunan Normal University, Changsha, Hunan 410081, China
[5] School of Public Policy and Administration, Xi'an Jiaotong University, Xi'an 710049, China; liuqiuyu@xjtu.edu.cn (Q.L.)
[6] Hardwood Tree Improvement and Regeneration Center, Department of Forestry and Natural Resources, Purdue University, West Lafayette, IN 47907, USA; djacobs@purdue.edu (D.F.J.)
[7] School for Forest Management, Swedish University of Agricultural Sciences, Skinnskatteberg 739 21, Sweden
* Correspondence: fenghuili@hainanu.edu.cn

This file includes: Figure S1 to S4
Tables S1 to S13

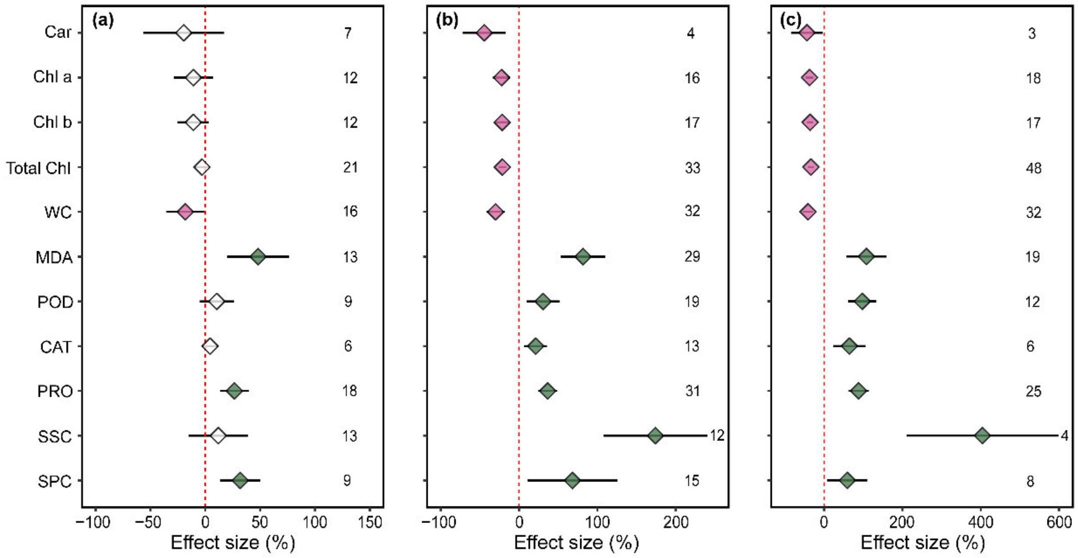

Figure S1. Effect size of drought on physiological traits under low (a), moderate (b), and severe (c) drought intensities in ferns. Diamonds show mean effect sizes, and black lines show 95% confidence intervals. The red dashed line marks effect size = 0. Green symbols indicate significant positive effects, pink symbols indicate significant negative effects, and white symbols indicate nonsignificant effects. Numbers indicate independent observations. Car, carotenoid content; Chl a, chlorophyll a; Chl b, chlorophyll b; Total Chl, total chlorophyll; WC, water content; MDA, malondialdehyde; POD, peroxidase; CAT, catalase; PRO, proline; SSC, soluble sugar; SPC, soluble protein.

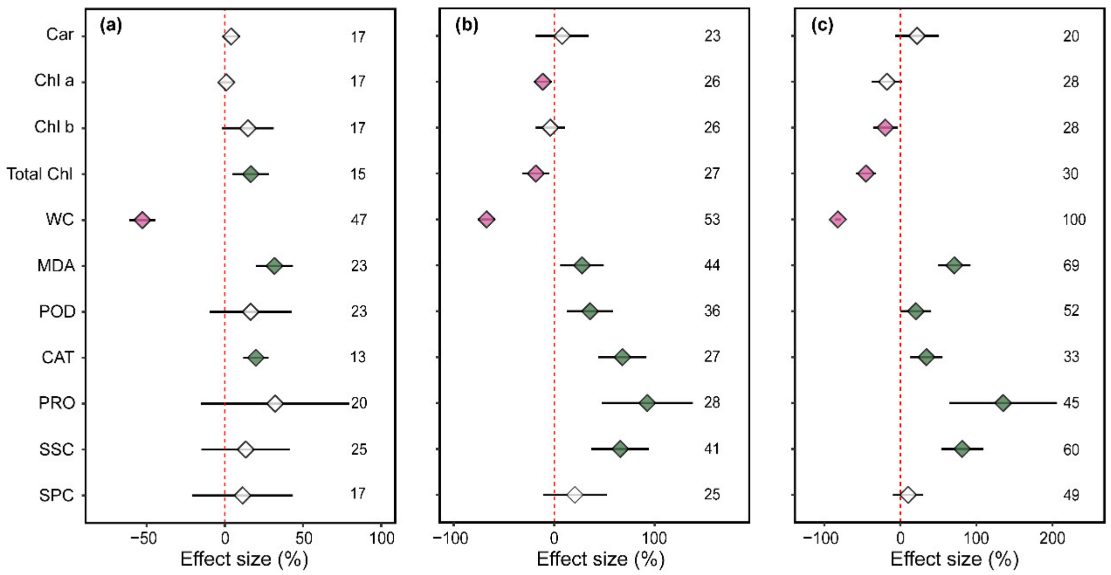

Figure S2. Effect size of drought on physiological traits under low (a), moderate (b), and severe (c) drought intensities in mosses. Diamonds show mean effect sizes, and black lines show 95% confidence intervals. The red dashed line marks effect size = 0. Green symbols indicate significant positive effects, pink symbols indicate significant negative effects, and white symbols indicate nonsignificant effects. Numbers indicate independent observations. Car, carotenoid content; Chl a, chlorophyll a; Chl b, chlorophyll b; Total Chl, total chlorophyll; WC, water content; MDA, malondialdehyde; POD, peroxidase; CAT, catalase; PRO, proline; SSC, soluble sugar; SPC, soluble protein.

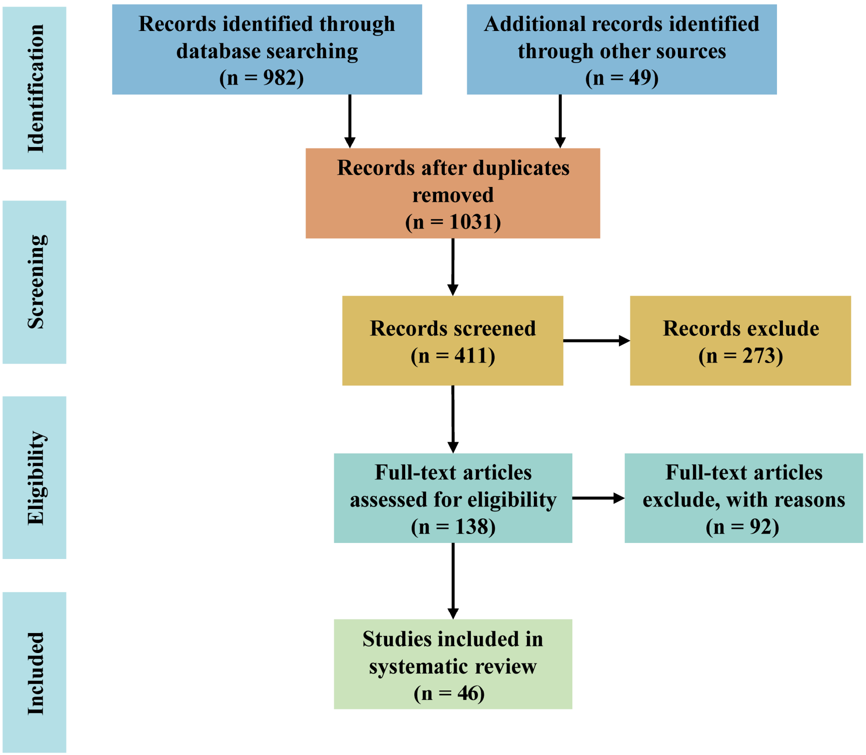

Figure S3. Preferred reporting items for systematic reviews and meta-analyses (PRISMA) flowchart illustrating the study selection process for this meta-analysis. The numbers (n) at each stage in the diagram represent the number of literature records or articles included or excluded during the screening process.

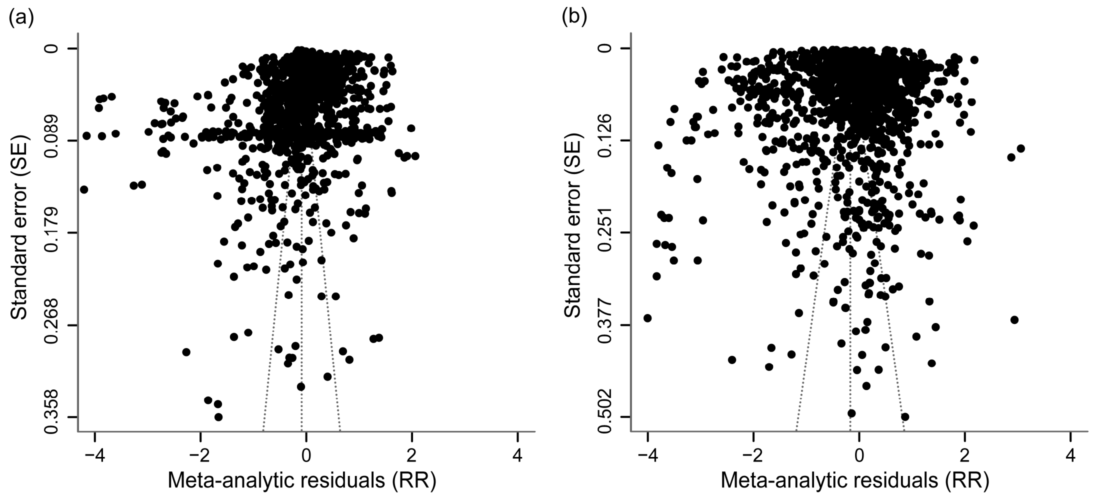

Figure S4. Funnel plots showing the relationship between meta-analytic residuals and standard error are used to detect funnel plot asymmetry for ferns dataset (a) and mosses dataset (b).

Table S1. Summary of the results of the meta-analysis on the response of drought on the physiological traits of ferns and mosses at a global scale, using the response metric *RR* (%).

Response object	N	Mean	LCI	UCI	*P*-value
Ferns	1463	–8.3	–11.7	–4.9	**< 0.0001**
Mosses	1808	–15.4	–19.6	–11.3	**< 0.0001**

LCI and UCI represent the lower and upper bounds of the 95% confidence intervals. N represent the sample sizes. Bold values are significant ($P < 0.05$).

Table S2. Summary of the results of the meta-analysis on the response of drought to the key indicators of plant photosynthetic efficiency, pigment composition, and water relations of ferns at a global scale, using the response metric *RR* (%).

RR	N	Mean	LCI	UCI	*P*-value
Car	14	–40.4	–60.4	–20.4	**0.0012**
Chl a	46	–26.5	–33.9	–19.0	**< 0.0001**
Chl b	46	–25.2	–31.4	–18.9	**< 0.0001**
Chl a/b	41	–2.4	–6.2	1.4	0.2070
Total Chl	102	–24.0	–28.8	–19.2	**< 0.0001**
NDCI	26	–9.7	–12.8	–6.7	**< 0.0001**
qP	45	1.5	–1.1	4.1	0.2634
NPQ	45	93.8	71.7	115.8	**< 0.0001**
Φ_{PSII}	45	–3.1	–8.0	1.9	0.2153
F_v	15	–10.6	–16.1	–5.1	**0.0003**
F_m	45	–3.3	–15.5	8.8	0.5175
F_0	45	–27.2	–37.7	–16.7	**< 0.0001**
F_v/F_m	84	9.3	–5.6	24.2	0.2557
g_s	89	–54.7	–62.6	–46.8	**< 0.0001**
C_i	26	42.4	30.9	53.9	**< 0.0001**
TR	36	–77.6	–85.9	–69.2	**< 0.0001**
P_n	12	–31.6	–43.3	–20.0	**< 0.0001**
P_{max}	2	–22.6	–38.3	–7.0	**0.0079**
A_{max}	18	–79.3	–93.7	–64.9	**< 0.0001**
WC	80	–33.5	–41.6	–25.3	**< 0.0001**
WBI	7	–7.8	–11.2	–4.4	**< 0.0001**

LCI and UCI represent the lower and upper bounds of the 95% confidence intervals. N represent the sample sizes. Car, carotenoid content; Chl a, chlorophyll a; Chl b, chlorophyll b; Chl a/b, chlorophyll a to b ratio; Total Chl, total chlorophyll; NDCI, normalized difference chlorophyll index; *qP*, photochemical quenching; NPQ, non-photochemical quenching; Φ_{PSII}, effective quantum yield of photosystem II; F_v, variable fluorescence; F_m, maximal fluorescence; F_0, minimum fluorescence; F_v/F_m, maximal quantum yield of photosystem II; g_s, stomatal conductance; C_i, intercellular carbon dioxide concentration; TR, transpiration rate; P_n, net photosynthetic rate; P_{max}, maximum photosynthetic rate; A_{max}, maximum net photosynthetic rate; WC, water content; WBI, water band index. Bold values are significant ($P < 0.05$).

Table S3. Summary of the results of the meta-analysis on the response of drought to the key indicators of plant photosynthetic efficiency, pigment composition, and water relations of mosses at a global scale, using the response metric *RR* (%).

RR	N	Mean	LCI	UCI	*P*-value
Car	60	10.2	–3.3	23.7	0.1521
Chl a	71	–12.0	–21.4	–2.6	**0.0149**
Chl b	71	–7.7	–17.7	2.4	0.1237
Total Chl	72	–25.7	–35.9	–15.5	**< 0.0001**
qP	20	–7.1	–11.5	–2.6	**0.0024**
NPQ	20	99.9	59.8	140.0	**< 0.0001**
Φ_{PSII}	130	–20.8	–25.6	–16.1	**< 0.0001**
F_m	20	–25.2	–32.4	–17.9	**< 0.0001**
F_0	20	30.9	20.4	41.4	**< 0.0001**
F_v/F_m	37	–21.6	–30.1	–13.2	**< 0.0001**
TR	15	–20.1	–28.9	–11.2	**< 0.0001**
P_n	15	–63.8	–79.4	–48.3	**< 0.0001**
P_{max}	8	–60.3	–76.8	–43.8	**< 0.0001**
WUE	15	–46.7	–58.3	–35.2	**< 0.0001**
WC	200	–73.9	–77.7	–70.2	**< 0.0001**
WBI	110	–18.0	–19.6	–16.4	**< 0.0001**

LCI and UCI represent the lower and upper bounds of the 95% confidence intervals. N represent the sample sizes. Car, carotenoid content; Chl a, chlorophyll a; Chl b, chlorophyll b; Total Chl, total chlorophyll; *qP*, photochemical quenching; NPQ, non-photochemical quenching; Φ_{PSII}, effective quantum yield of photosystem II; F_m, maximal fluorescence; F_0, minimum fluorescence; F_v/F_m, maximal quantum yield of photosystem II; TR, transpiration rate; P_n, net photosynthetic rate; P_{max}, maximum photosynthetic rate; WUE, water use efficiency; WC, water content; WBI, water band index. Bold values are significant ($P < 0.05$).

Table S4. Summary of the results of the meta-analysis on the response of drought to the osmoregulatory substances, hormone regulation, and growth strategy parameters of ferns at a global scale, using the response metric *RR* (%).

RR	N	Mean	LCI	UCI	*P*-value
H_2O_2	8	67.3	–34.1	168.6	0.4255
MDA	61	79.1	58.1	100.0	**< 0.0001**
RC	39	137.1	102.2	171.9	**< 0.0001**
SOD	60	13.9	1.2	26.5	**0.0298**
POD	40	40.9	23.9	57.9	**< 0.0001**
CAT	25	23.1	9.5	36.7	**0.0004**
APX	8	–1.0	–38.4	36.3	0.6669
TFC	16	21.1	–1.9	44.2	0.0783
TPC	16	5.0	–11.6	21.6	0.6562
PRO	74	48.8	37.9	59.6	**< 0.0001**
SSC	29	98.8	49.0	148.6	**< 0.0001**
SPC	32	50.5	22.6	78.4	**< 0.0001**
FAA	8	–17.9	–41.0	5.3	0.1067
ABA	8	–20.1	–35.2	–5.0	**0.0128**
R:S	14	8.4	–1.4	18.3	0.0997
AGB	10	–11.3	–15.4	–7.2	**< 0.0001**
BGB	22	–9.1	–15.3	–3.0	**0.0045**
LB	18	–15.0	–25.3	–4.7	**0.0064**
SLA	26	9.6	3.0	16.1	**0.0034**
LAR	54	–40.3	–46.5	–34.1	**< 0.0001**

LCI and UCI represent the lower and upper bounds of the 95% confidence intervals. N represent the sample sizes. H_2O_2, hydrogen peroxide; MDA, malondialdehyde; RC, relative conductivity; SOD, superoxide dismutase; POD, peroxidase; CAT, catalase; APX, ascorbate peroxidase; TFC, total flavonoids; TPC, total phenols; PRO, proline; SSC, soluble sugar; SPC, soluble protein; FAA, free amino acids; ABA, abscisic acid; R:S, root to shoot ratio; AGB, aboveground biomass; BGB, belowground biomass; LB, leaf biomass; SLA, specific leaf area; LAR, leaf area ratio. Bold values are significant ($P < 0.05$).

Table S5. Summary of the results of the meta-analysis on the response of drought to the osmoregulatory substances, hormone regulation, and growth strategy parameters of mosses at a global scale, using the response metric *RR* (%).

RR	N	Mean	LCI	UCI	*P*-value
H_2O_2	7	28.4	16.5	40.2	**< 0.0001**
RO_2^-	15	162.2	92.3	232.0	**< 0.0001**
MDA	136	47.2	33.9	60.4	**< 0.0001**
MP	30	86.0	47.8	124.3	**< 0.0001**
RC	21	91.6	66.8	116.4	**< 0.0001**
SOD	100	25.2	11.3	39.0	**0.0001**
POD	111	23.0	9.9	36.1	**0.0002**
CAT	73	42.1	28.9	55.4	**< 0.0001**
APX	3	38.2	27.8	48.6	**< 0.0001**
PRO	93	90.2	54.6	125.8	**< 0.0001**
SSC	126	58.6	41.4	75.8	**< 0.0001**
SPC	91	11.2	–3.6	26.1	0.1533
FAA	16	58.3	30.1	86.5	**< 0.0001**
ABA	9	81.0	19.6	142.4	**0.0032**
AGB	10	–86.4	–88.8	–84.1	**< 0.0001**
MSR	80	–90.6	–94.6	–86.6	**< 0.0001**

LCI and UCI represent the lower and upper bounds of the 95% confidence intervals. N represent the sample sizes. H_2O_2, hydrogen peroxide; RO_2^-, superoxide generating rate; MDA, malondialdehyde; MP, membrane permeability; RC, relative conductivity; SOD, superoxide dismutase; POD, peroxidase; CAT, catalase; APX, ascorbate peroxidase; PRO, proline; SSC, soluble sugar; SPC, soluble protein; FAA, free amino acids; ABA, abscisic acid; AGB, aboveground biomass; MSR, mean survival rate. Bold values are significant ($P < 0.05$).

Table S6. Summary of the results of the meta-analysis on the response of drought to epiphytic ferns across ecological habits at a global scale, using the response metric *RR* (%).

RR	N	Mean	LCI	UCI	*P*-value
Terrestrial	969	3.2	–0.4	6.8	0.0832
Epiphytic	494	–27.3	–33.2	–21.3	**< 0.0001**
Obligate	387	–33.8	–40.1	–27.6	**< 0.0001**
Facultative	107	3.3	–12.0	18.6	0.7804
Tank-forming	276	–36.1	–43.3	–28.8	**< 0.0001**
Non-tank-forming	218	–13.9	–23.7	–4.1	**0.0073**
Canopy	338	–38.5	–44.8	–32.2	**< 0.0001**
Understory	156	5.1	–6.7	16.8	0.4508
Hydrophilic	309	–12.1	–17.3	–6.9	**< 0.0001**
Xerophytic	185	–46.4	–56.3	–36.5	**< 0.0001**

LCI and UCI represent the lower and upper bounds of the 95% confidence intervals. N represent the sample sizes. Bold values are significant ($P < 0.05$).

Table S7. Summary of the results of the meta-analysis on the response of drought under low drought intensities in ferns at a global scale, using the response metric *RR* (%).

RR	N	Mean	LCI	UCI	*P*-value
Car	7	–19.6	–56.3	17.1	0.1826
Chl a	12	–10.8	–28.7	7.1	0.1931
Chl b	12	–10.9	–25.1	3.3	0.1174
Total Chl	21	–3.0	–9.7	3.8	0.3593
WC	16	–18.2	–35.5	–0.9	**0.0409**
MDA	13	48.2	20.0	76.3	**0.0001**
POD	9	10.5	–5.2	26.2	0.2176
CAT	6	4.4	–2.9	11.6	0.2564
PRO	18	26.6	13.7	39.4	**< 0.0001**
SSC	13	11.9	–15.4	39.1	0.5201
SPC	9	31.8	13.8	49.9	**0.0001**

LCI and UCI represent the lower and upper bounds of the 95% confidence intervals. N represent the sample sizes. Car, carotenoid content; Chl a, chlorophyll a; Chl b, chlorophyll b; Total Chl, total chlorophyll; WC, water content; MDA, malondialdehyde; POD, peroxidase; CAT, catalase; PRO, proline; SSC, soluble sugar; SPC, soluble protein. Bold values are significant ($P < 0.05$).

Table S8. Summary of the results of the meta-analysis on the response of drought under moderate drought intensities in ferns at a global scale, using the response metric *RR* (%).

RR	N	Mean	LCI	UCI	*P*-value
Car	4	–44.5	–72.1	–16.9	**0.0087**
Chl a	16	–22.3	–33.2	–11.5	**0.0003**
Chl b	17	–21.6	–30.1	–13.1	**< 0.0001**
Total Chl	33	–21.3	–26.2	–16.4	**< 0.0001**
WC	32	–30.0	–41.5	–18.5	**< 0.0001**
MDA	29	81.7	53.0	110.4	**< 0.0001**
POD	19	30.7	9.8	51.5	**0.0019**
CAT	13	21.3	6.8	35.8	**0.0024**
PRO	31	36.6	24.7	48.4	**< 0.0001**
SSC	12	174.0	108.0	240.0	**< 0.0001**
SPC	15	68.2	11.1	125.2	**0.011**

LCI and UCI represent the lower and upper bounds of the 95% confidence intervals. N represent the sample sizes. Car, carotenoid content; Chl a, chlorophyll a; Chl b, chlorophyll b; Total Chl, total chlorophyll; WC, water content; MDA, malondialdehyde; POD, peroxidase; CAT, catalase; PRO, proline; SSC, soluble sugar; SPC, soluble protein. Bold values are significant ($P < 0.05$).

Table S9. Summary of the results of the meta-analysis on the response of drought under severe drought intensities in ferns at a global scale, using the response metric *RR* (%).

RR	N	Mean	LCI	UCI	*P*-value
Car	3	–43.9	–84.4	–3.4	**0.042**
Chl a	18	–37.1	–47.0	–27.2	**< 0.0001**
Chl b	17	–35.6	–43.5	–27.8	**< 0.0001**
Total Chl	48	–33.7	–40.8	–26.5	**< 0.0001**
WC	32	–41.1	–54.4	–27.8	**< 0.0001**
MDA	19	108.1	57.4	158.7	**< 0.0001**
POD	12	97.4	61.6	133.2	**< 0.0001**
CAT	6	64.6	23.9	105.2	**0.0003**
PRO	25	88.0	62.5	113.5	**< 0.0001**
SSC	4	404.2	210.5	598.0	**< 0.0001**
SPC	8	59.1	7.2	111.1	**0.0182**

LCI and UCI represent the lower and upper bounds of the 95% confidence intervals. N represent the sample sizes. Car, carotenoid content; Chl a, chlorophyll a; Chl b, chlorophyll b; Total Chl, total chlorophyll; WC, water content; MDA, malondialdehyde; POD, peroxidase; CAT, catalase; PRO, proline; SSC, soluble sugar; SPC, soluble protein. Bold values are significant ($P < 0.05$).

Table S10. Summary of the results of the meta-analysis on the response of drought under low drought intensities in mosses at a global scale, using the response metric *RR* (%).

RR	N	Mean	LCI	UCI	*P*-value
Car	17	4.1	–1.6	9.7	0.1657
Chl a	17	1.0	–3.7	5.6	0.7132
Chl b	17	14.8	–1.7	31.3	0.0834
Total Chl	15	16.6	4.9	28.3	**0.0037**
WC	47	–52.7	–61.1	–44.4	**<0.0001**
MDA	23	31.7	19.9	43.5	**<0.0001**
POD	23	16.5	–9.8	42.8	0.2802
CAT	13	19.9	11.8	28.0	**<0.0001**
PRO	20	32.3	–15.2	79.8	0.2721
SSC	25	13.4	–15.0	41.8	0.4747
SPC	17	11.4	–20.8	43.5	0.6716

LCI and UCI represent the lower and upper bounds of the 95% confidence intervals. N represent the sample sizes. Car, carotenoid content; Chl a, chlorophyll a; Chl b, chlorophyll b; Total Chl, total chlorophyll; WC, water content; MDA, malondialdehyde; POD, peroxidase; CAT, catalase; PRO, proline; SSC, soluble sugar; SPC, soluble protein. Bold values are significant ($P < 0.05$).

Table S11. Summary of the results of the meta-analysis on the response of drought under moderate drought intensities in mosses at a global scale, using the response metric *RR* (%).

RR	N	Mean	LCI	UCI	*P*-value
Car	23	7.9	–18.4	34.2	0.7216
Chl a	26	–11.3	–20.1	–2.6	**0.0132**
Chl b	26	–3.8	–18.4	10.8	0.5166
Total Chl	27	–18.3	–31.4	–5.1	**0.0094**
WC	53	–67.2	–75.8	–58.7	**< 0.0001**
MDA	44	27.7	6.0	49.4	**0.0088**
POD	36	35.7	12.8	58.6	**0.0008**
CAT	27	67.8	43.8	91.8	**< 0.0001**
PRO	28	92.6	47.1	138.0	**< 0.0001**
SSC	41	65.7	37.1	94.4	**< 0.0001**
SPC	25	20.7	–11.0	52.4	0.2675

LCI and UCI represent the lower and upper bounds of the 95% confidence intervals. N represent the sample sizes. Car, carotenoid content; Chl a, chlorophyll a; Chl b, chlorophyll b; Total Chl, total chlorophyll; WC, water content; MDA, malondialdehyde; POD, peroxidase; CAT, catalase; PRO, proline; SSC, soluble sugar; SPC, soluble protein. Bold values are significant ($P < 0.05$).

Table S12. Summary of the results of the meta-analysis on the response of drought under severe drought intensities in mosses at a global scale, using the response metric *RR* (%).

RR	N	Mean	LCI	UCI	*P*-value
Car	20	22.1	–6.6	50.7	0.1618
Chl a	28	–17.4	–37.6	2.7	0.0799
Chl b	28	–19.7	–35.9	–3.6	**0.0207**
Total Chl	30	–45.1	–58.3	–32.0	**< 0.0001**
WC	100	–82.0	–85.9	–78.2	**< 0.0001**
MDA	69	71.0	49.6	92.4	**< 0.0001**
POD	52	20.4	0.3	40.6	**0.0466**
CAT	33	34.4	13.2	55.6	**0.0005**
PRO	45	135.2	64.4	206.1	**< 0.0001**
SSC	60	81.3	53.8	108.8	**< 0.0001**
SPC	49	10.3	–9.7	30.3	0.3849

LCI and UCI represent the lower and upper bounds of the 95% confidence intervals. N represent the sample sizes. Car, carotenoid content; Chl a, chlorophyll a; Chl b, chlorophyll b; Total Chl, total chlorophyll; WC, water content; MDA, malondialdehyde; POD, peroxidase; CAT, catalase; PRO, proline; SSC, soluble sugar; SPC, soluble protein. Bold values are significant ($P < 0.05$).

Table S13. Summary of global meta-analysis of the effects of drought on the physiological traits in ferns and mosses.

Response object	k	τ^2	Q_t	*P-value* (Q_t)	I^2
Ferns	1463	0.52	670,032.4	**< 0.0001**	99.94%
Mosses	1808	1.12	2,425,846.3	**< 0.0001**	99.98%

k is the number of effect sizes; τ^2 represent study-level variance; Q_t is the total heterogeneity of effect sizes; I^2 is metric quantifying the heterogeneity to sampling variance.
